# Hepatocyte Growth Factor: A Microenvironmental Resource for Leukemic Cell Growth

**DOI:** 10.3390/ijms20020292

**Published:** 2019-01-12

**Authors:** Paolo Giannoni, Franco Fais, Giovanna Cutrona, Daniela de Totero

**Affiliations:** 1 Stem Cell Laboratory, Department of Experimental Medicine, University of Genoa, V. Pastore 3, 16132 Genova, Italy; paolo.giannoni@unige.it; 2 Molecular Pathology Unit, IRCCS Polyclinic Hospital San Martino, L.go R. Benzi n.10, 16132 Genova, Italy; franco.fais@unige.it (F.F.); giovanna.cutrona@hsanmartino.it (G.C.)

**Keywords:** HGF, c-MET, hematological neoplasia, microenvironment

## Abstract

Chronic lymphocytic leukemia (CLL) is characterized by the progressive expansion of B lymphocytes CD5+/CD23+ in peripheral blood, lymph-nodes, and bone marrow. The pivotal role played by the microenvironment in disease pathogenesis has become increasingly clear. We demonstrated that bone marrow stromal cells and trabecular bone cells sustain survival of leukemic B cells through the production of hepatocyte growth factor (HGF). Indeed the trans-membrane kinase receptor for HGF, c-MET, is expressed on CLL cells and STAT3 TYR^705^ or AKT phosphorylation is induced after HGF/c-MET interaction. We have further observed that c-MET is also highly expressed in a peculiar type of cells of the CLL-microenvironment showing nurturing features for the leukemic clone (nurse-like cells: NLCs). Since HGF treatment drives monocytes toward the M2 phenotype and NLCs exhibit features of tumor associated macrophages of type 2 we suggested that HGF, released either by cells of the microenvironment or leukemic cells, exerts a double effect: (i) enhances CLL cells survival and (ii) drives differentiation of monocytes-macrophages to an oriented immune suppressive phenotype. We here discuss how paracrine, but also autocrine production of HGF by malignant cells, may favor leukemic clone expansion and resistance to conventional drug treatments in CLL, as well as in other hematological malignancies. Novel therapeutic approaches aimed to block HGF/c-MET interactions are further proposed.

## 1. Introduction

Chronic lymphocytic leukemia (CLL), the most common leukemia in the western world, is characterized by a progressive expansion of relative mature B lymphocytes, CD19+/CD5+/CD23+, in peripheral blood, secondary lymphoid tissues, and in bone marrow (BM) [1]. To date, CLL is still an incurable disease although novel therapies inhibiting kinases activated by BCR triggering (Bruton Tyrosine Kinase: BTK, Spleen Tyrosine Kinase: SYK, Phospho-Inositol-3-phosphate Kinase: PI3K) have significantly improved overall survival [2,3].

Chronic lymphocytic leukemia cells in vivo show an apparent long half-life while they tend to rapidly undergo apoptosis when cultured in vitro, in medium supplemented with only fetal bovine serum. This observation suggests that prolonged in vivo survival may be due to cell-to-cell interactions or soluble factors, present in the microenvironment, protecting leukemic cells from apoptotic cell death [4,5,6,7,8,9]. Therefore, the attempt of disrupting protective signals provided by the CLL microenvironment represents an attractive avenue to develop novel therapeutic strategies.

The interaction between hepatocyte growth factor (HGF) and its receptor c-MET regulates various physiological processes such as embryogenesis, regeneration of damaged organs, hematopoiesis, and immune cell functions. However the aberrant expression of c-MET and of HGF is implicated in neoplastic cells spreading and tumor progression in many solid tumors [10,11,12,13].

To date, only a few studies have addressed the role of HGF in lymphomas. Moreover data from these reports, on the supposed relationship between enhanced c-MET expression and disease pathogenesis, are sometimes conflicting, especially in diffuse large B cell lymphoma (DLBCL) or in Hodgkin disease (HD) [14,15]. Numerous studies have instead clearly established a key role for the HGF/c-MET axis in promoting myeloma cells aggressiveness. Indeed, the prognosis of patients with multiple myeloma (MM) is worse in the presence of high HGF levels in serum and a direct correlation between serum HGF levels and disease aggressiveness has been demonstrated [16,17,18,19]. Moreover, the levels of c-MET expression in plasma-cells (PCs) of MM patients may predict response to therapy: PCs overexpressing c-MET characterize a sub-group of patients showing low response to therapy and poor clinical outcome [20].

From our studies, first focused to clarify whether various cell types sharing the same embryonic origin (bone marrow stromal cells, osteoblasts, fibroblasts) affect survival and expansion of the leukemic B cell clone, we identified HGF as a novel factor, released from cells of the CLL-microenvironment, capable of protecting leukemic cells from apoptosis [21]. We have further demonstrated that HGF contributes to modify features of monocytes in CLL, driving them toward an alternative and immunosuppressive M2 phenotype. Moreover we observed that leukemic B cells start to produce HGF after their co-culture with nurse-like cells (NLCs), a peculiar type of tumor associated monocytes (TAM) present in this disease.

We here describe how the HGF/c-MET axis is involved in a complex scenario taking place in different hematological malignancies: we will deepen details relative to the CLL microenvironment to explicit how HGF, produced within the stroma by different mesenchymal cell types and by the CLL cells themselves, contributes to increase viability of the c-MET+ leukemic clone and further affects differentiation of surrounding cells, leading to suppression of normal immune responses. Moreover, we will summarize and explore therapies aimed to block survival advantages provided by c-MET/HGF interactions in leukemia.

## 2. HGF and c-MET Expression and Function in Hematological Neoplasms

HGF first discovered as a potent stimulator of cultured hepatocytes [22] is produced as a one-chain inactive pro-protein, later cleaved into two biologically active chains by plasmatic enzymes such as HGF activator (HGFA), thrombin, type II transmembrane enzyme matryptase, hepsin, and uPAR [23]. The HGF receptor c-MET, a receptor tyrosine kinase, is composed of a 50 KDa extracellular subunit linked to a 145 KDa chain by a disulphide bond. Binding of biologically active HGF to c-MET induces receptor dimerization and trans-autophosphorylation at residues Y1234/Y1235 in the activation loop of the kinase domain. Subsequent phosphorylation of the two docking tyrosines, Y1249 and Y1356, in the carboxy-terminal multifunctional docking region recruits several adaptors molecules and transduces downstream signaling pathways such as GRB2, SOS, RAS, RAF, MEK, or ERK. Hepatocyte growth factor/c-MET interaction thus triggers a variety of signaling pathways leading to increased cell proliferation, migration, invasion, angiogenesis and transformation [13].

c-MET is largely expressed on epithelial cells but certain hematopoietic cells may also carry this receptor and gain sensitivity to HGF: c-MET is present in B cells of the germinal center or in terminally differentiated plasma-cells [24]. In normal conditions hematopoietic progenitors do not produce HGF, while bone marrow stromal cells (BMSC) are a physiological source of this cytokine. Therefore, in physiological conditions, the action of HGF on hematopoietic progenitors is paracrine. However in some malignancies such as in Philadelphia chromosome negative and positive (Ph− or Ph+) myeloproliferative neoplasms (MPNs) or in multiple myeloma (MM) HGF derives from cells of the microenvironment but also from leukemic cells or their progenitors. Since CD34+ progenitors from patients with Ph+ MPN or with myelodisplastic syndrome (MDS) show aberrant c-MET expression on their membrane, an autocrine loop of activation of the HGF/c-MET pathway should be envisaged [16]. HGF expression, leading to autocrine activation of c-MET, was further observed in nearly half of AML cell lines and in samples from AML patients [25]. Interestingly, HGF seems also to be one of the most differentially expressed genes in the leukemia-initiating cells of AML, as compared to normal hematopoietic stem cells [26]. c-Met expression on leukemic cells, as well as plasmatic HGF concentration, is also increased in patients with acute T lymphocytic lymphoma (ATLL), while either of them is rarely found in subjects suffering of chronic disease. Therefore, in aggressive form of ATLL, a paracrine as well as an autocrine release of HGF may stimulate the growth of leukemic cells [27,28]. c-MET and HGF are expressed in natural killer/ T cell lymphoma cell lines and in nasal NK/T cell lymphoma specimens thus suggesting that also NK/T cell lymphoma proliferation may be induced through an autocrine loop [29].

The HGF/c-MET pathway appears further largely activated in malignant cells from primary effusion lymphoma (PEL): here the expression of plexin B1 is required for c-MET-mediated survival of PEL cells. Interestingly, targeting of c-MET/HGF pathway successfully suppresses PEL diffusion in a pre-clinical mouse model [30,31,32]. c-MET is frequently overexpressed in diffuse large B cell lymphoma (DLBCL), occasionally present in Burkitt or in follicular lymphomas (BL, FL) [33,34] but negative in mantle cell (MCL) or marginal zone lymphoma. It is however still unclear whether high c-MET expression in DLBCL could correlate with poor prognosis: indeed, high c-MET expression on malignant cells has been alternatively associated with unfavorable [35] or better prognosis [36,37]. To provide explanations for these conflicting and unexpected results Uddin et al. have suggested that c-MET+ leukemic cells, showing higher proliferation rates and higher Ki67 positivity, may be more responsive to chemotherapy. In agreement with this hypothesis previous studies have shown, in other types of NHLs, that tumors characterized by a higher number of proliferating cells respond better to chemotherapy [38]. The same authors have demonstrated that in vitro treatment with the specific c-MET tyrosine kinase inhibitor PHA665752, induced apoptosis of DLBCL cells through inhibition of the PI3K/AKT pathway: based on these observations they suggested that c-MET targeting may thus represent a valuable therapy for DLBCL [37]. Moreover, it is worth noting that c-MET overexpression in DLBCL appears more common in the GC subtype than in the ABC subtype [37] and that c-MET is consistently increased upon transformation of low grade FL into DLBCL [39]. Interestingly high amounts of HGF have been detected in sera from DLBCL patients and these levels are directly correlated with clinical outcome and bad prognosis [35,40,41]. It has been observed that HGF, within the DLBCL microenvironment, is produced by activated macrophages, while leukemic cells produce only the HGF activator protease (HGFA). Hence, autocrine production of HGFA by DLBCL cells may support tumorigenesis via autocatalyzation, thus providing a constant source of active HGF within the microenvironment [34].

Multiple studies have established that the HGF/c-MET pathway may have a critical role in MM pathogenesis: elevated HGF and HGFA concentrations were in fact detected in sera from MM patients and were correlated with progressive disease [42,43]. Furthermore, successful responses to therapies paralleled a consistent decrease in HGF levels in sera patients [44]. An association between HGF gene expression in BM samples and the extent of lytic bone disease in MM patients has been also documented [45]: HGF, present at high levels in MM patients, inhibits in fact osteoblastic differentiation thus subsequently reducing bone formation rates and contributing to myeloma bone disease [46]. It has been further observed that surrounding stromal cells of the BM microenvironment stimulate the release of HGF from MM cells: MM cells purified and cultured in vitro with BMSCs start to produce HGF [47,48,49]. Immunohistochemistry analyses of bone marrow biopsies from MM patients, as compared to normal donors, have further demonstrated that HGF and c-MET are concomitantly expressed in plasma cells. Membranous staining of c-MET was found in 40% of the MM samples but in none of the 10 healthy individuals analyzed. Phospho-c-MET was further confined to malignant plasma cells, as opposed to normal plasma cells, thus pointing to activation of the HGF/c-MET pathway in MM patients in vivo [50]. Endothelial cells present in BM from MM patients also show higher HGF production and higher c-MET expression than endothelial cells from normal BM controls, thus indicating a role of autocrine HGF/c-MET activation in MM angiogenesis [17].

HGF and c-MET clearly contribute to MM pathogenesis, while data reported from various authors about a potential relationship between prognosis and c-MET expression in Hodgkin lymphomas are instead sometimes discordant [51,52,53]. Teofili et al. have observed positivity for c-MET but not for HGF on Reed Stemberg cells (RS), independently of the presence of EBV infection [52]. Moreover several reactive dendritic-reticulum cells showed HGF positivity while RS cells expressed only the α4 and α5 integrins, specifically activated by HGF to promote cell adhesion. Altogether, these findings suggest the involvement of the HGF/c-MET pathway in favoring expansion of neoplastic cells and in disease pathogenesis. Bedewy et al. [54] have further observed high c-MET expression in more than 30% of cells within HD tissues in 56% of patients analyzed: c-MET levels were also associated with adverse prognostic parameters, such as older age at diagnosis, leukocytosis, presence of B symptoms and lower chance of complete remission. In contrast to these suggestions is the report from Xu et al. which described a direct correlation between c-MET overexpression and favorable prognosis. However the results obtained from the same authors in in vitro experiments appear in conflict with clinical data: they in fact demonstrated that the c-MET inhibitor SU11274, in different HD cell lines, blocked constitutive c-MET phosphorylation and induced cell cycle arrest at G2/M [55]. 

This brief overview outlines how upregulated c-MET expression on leukemic cells, and paracrine but also autocrine release of HGF (Table 1), may create favorable conditions to the expansion of myeloid and lymphoid cells in hematological diseases. Data from different authors about a relationship between disease outcome and HGF or c-MET overexpression appear however discordant in some types of malignancies (i.e., DLBCL, HD), while for others, such as MM or AML and Ph+MPN, several reports have univocally confirmed a pivotal role of HGF/c-MET in pathogenesis. Further work might be thus of help to better extend, confirm, and clarify the above described findings.

## 3. Microenvironment as a Source of HGF in CLL

### 3.1. HGF Increases Viability of Leukemic Cells

The peripheral blood represents only part of the CLL life cycle. The observation that most circulating CLL cells are out of the cell cycle but have undergone a significant number of divisions, as demonstrated by in vivo kinetic studies with deuterated water labeling [57], suggested that accumulation of leukemic cells depends on the rate of cell birth and cell death: CLL cells, located in different districts, such as peripheral blood, lymph-nodes or bone marrow, receive different stimuli from their microenvironment. While circulating CLL cells appear non-proliferating, tissue-resident cells show instead high number of divisions. In CLL leukemic B cells proliferation takes place within particular lymph-nodes areas, the so called ‘proliferation centers’, where CLL cells receive specific hints to divide after their interactions with surrounding stromal cells, monocytes or T cells.

Herishanu et al. [58] through the comparison of gene expression profiles of CLL cells extracted from three different anatomic compartments (bone marrow, peripheral blood or lymph-node) have demonstrated that lymph-nodes represent the major site of CLL cells activation: here more than 100 genes, involved in BCR activation, NF-κB signaling or proliferation, were upregulated. The work from Herndon et al. [59] has also identified lymph-nodes as the anatomical site showing higher number of proliferating CLL cells which characteristically exhibit a CXCR4dim/CD5bright phenotype.

**Figure 1:**
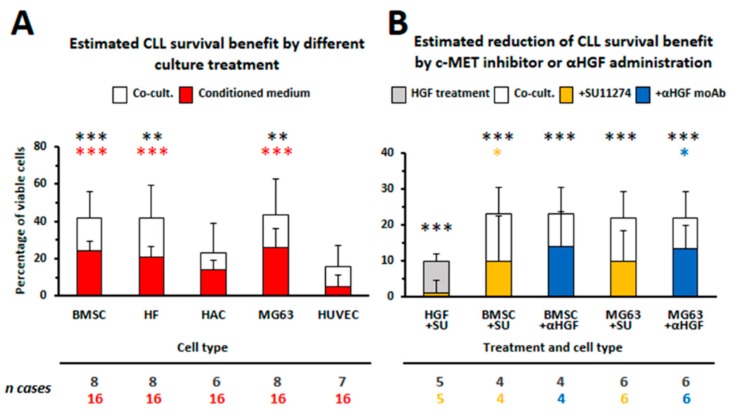
(**A**) Percentage of estimated CLL cells survival benefit due to conditioned medium or cell-to-cell contact (co-cultures). Depicted bars indicate the percentages of viable CLL cells deprived of relative control percentages in all experimental conditions, using the indicated different cell types (CTR co-cultures: 32.17 ± 31.62; CTR conditioned medium: 63 ± 22). The reported number of cases used (*n*) are color-linked to the relative bars and were used to generate the corresponding SD values. (**B**) Estimated reduction of CLL survival benefit due to addition of c-MET inhibitor or anti-HGF moAb in co-cultures or during exogenous HGF administration. Depicted bars indicate the percentages of viable CLL cells deprived of relative control percentages in all experimental conditions (CTR-HGF:70 ± 9.5: CTR-BMSC:58.8 ± 6.5; CTR-MG63:47.5 ± 22.9) using the indicated different cell types. The reported number of cases used (n) are color-linked to the relative bars and were used to generate the corresponding SD values. SU: SU11274, αHGF:HGF neutralizing moAb L2G7. Statistical significance (* *p* ≤ 0.05; **: 0.01 ≤ *p* < 0.001; ***: *p* ≤ 0.001; Student *t*-test) is referred to each culture condition in comparison to relative controls (CLL cells in medium only, here not shown as above described) and the present Figure is a different representation with minor modifications of previously published data reported by Giannoni P et al. in [21].

We first focused our attention to dissect whether different stromal components with the same mesenchymal origin may differentially sustain survival of CLL cells [21]. We determined that cell types prolonging leukemic cells survival, such as BMSCs, osteoblasts and fibroblasts, shared the release of two growth factors, CXCL12 and HGF, which were involved in protecting CLL cells from apoptosis [21]. The involvement of HGF, as a factor produced within the CLL-microenvironment and capable of increasing viability of leukemic cells, was novel (Figure 1). We demonstrated that CLL cells express the HGF receptor c-MET while a weak HGF mRNA expression was observed in some cases. Moreover only those cell types capable of protecting CLL cells from apoptosis show also mRNA expression for the HGF activator (HGFA), thus suggesting that an active HGF form should be available. When we determined the amounts of HGF released by different mesenchymal cells, we demonstrated that BMSCs, trabecular bone cells (TBMC) or the MG63 osteoblast-like cells line produced high amounts of HGF (range: 5000–10,000 pg/mL), whereas a much lower level was released by fibroblasts (HF; range: 50–100 pg/mL), as compared to the absence of release by endothelial cells or chondrocytes (HAC). Fibroblasts showed instead production of larger amounts of CXCL12 (10,000 pg/mL). Moreover by culturing CLL cells directly with exogenous HGF, we further provided the direct evidence of HGF pro-survival activity and demonstrated that HGF/c-MET interaction stimulated STAT3^TYR705^ and AKT phosphorylation. ERK1/2 appeared instead phosphorylated only after the addition of exogenous CXCL12 to CLL cells. These findings may apparently suggest that two different signaling pathways should be alternatively activated via HGF or via CXCL12, after CLL cells cross-talk with osteoblasts or with fibroblasts, respectively [21]. The involvement of HGF in enhancing CLL cells viability by stromal cells was further confirmed by silencing HGF transcripts in the osteoblast-like cell line MG63: spontaneous apoptosis appeared in fact restored after culturing CLL cells with HGF-silenced MG63 cells but not with mock-transfected MG63 cells [21]. Observations from Eksioglu-Demiralp et al. are also in agreement with our data [60]. They demonstrated higher expression of extracellular α and intracellular β chain of c-MET in CLL B cells than in B cells from normal controls. In addition HGF was higher in sera from CLL patients than in normal controls, induced CLL survival and increased the expression of survival signaling molecules such as PI3K/AKT, Bcl-XL and phospho- Bad^136^. Although we could not detect significant amounts of HGF in media from fresh CLL B cells cultured for 72 h, we instead found higher HGF levels in sera from CLL patients than in sera from normal controls (mean in CLL 932 ± 315 pg/mL, *n* = 10, vs mean in healthy controls, 601 ± 46 pg/mL, *n* = 3) as already reported [60,61]. These findings may suggest that high amounts of HGF, present in plasma from CLL patients, may derive from different cell types of the microenvironment but also from leukemic cells: as described subsequently, we determined that leukemic cells start to produce HGF only after their interaction with stromal microenvironmental cells.

### 3.2. CLL-Patient Monocytes Show Higher c-MET and IDO Expression than Controls Monocytes. HGF May Contribute to Drive Monocytes Toward an Immunosuppressive Phenotype

It has been described that HGF promotes monocyte differentiation toward the generation of cells producing IL-10 but not IL-12 and favors the expansion of T regulatory cells (Treg) [62,63]. When we analyzed the HGF receptor c-MET expression in monocytes from CLL patients, we observed that it was higher than in monocytes from normal donors. Through the myelo-monocyte THP1 cell line model we could further determine that HGF treatment upregulates the expression of c-MET as well as of Indoleamine 2,3-dyoxigenase (IDO), a key enzyme in the tryptophan catabolism that modulates T cell activation. IDO is further considered a hallmark of the M2 phenotype. We could also observe that THP1 cells, polarized toward the M2 phenotype (PMA+IL-4), showed higher c-MET, IDO, and TGFβ mRNA expression than THP1 cells polarized toward the M1 phenotype (PMA+IFNγ/LPS) [56]. We further determined that normal monocytes acquired features of M2 cells after their co-culture with CLL cells but not with normal B lymphocytes: the co-culture of purified monocytes from healthy donors with CLL B cells induced IDO and c-MET upregulation. These findings appear of particular relevance because suggest that neoplastic B cells, through the release of soluble factors such as HGF, may shape microenvironmental cells finally impairing their immune-competence. In this context we were prompted to characterize features of a peculiar type of cells of the CLL microenvironment named ‘nurse-like cells’ (NLCs) for their leukemic B cells nurturing function. Nurse-like cells, derived in vitro from cultures of mononuclear cells from CLL patients, express myelo-monocyte antigens although appear distinct from monocyte-macrophages or monocyte-derived dendritic cells [64,65]. Many evidences have supported the hypothesis that NLCs differentiate under the influence of leukemic CLL cells [65,66]. In vivo NLCs are present in the lymph-nodes of CLL patients, where they are interspersed with stromal, dendritic or T cells to form proliferation centers and where they may receive stimuli from microenvironment components as well as from the accumulating neoplastic B cells [4]. We have demonstrated that NLCs, derived in vitro, show high expression of c-MET, IL-10 and IDO, and that HGF stimulation induces STAT3^TYR705^ phosphorylation. More importantly, we further confirmed c-MET and IDO positivity, through immune-histochemical staining, in cells resembling NLCs morphology within BM and lymph-nodes biopsies from CLL patients. Moreover, in vivo, IDO appeared co-expressed with CD163, an antigen typically displayed by tumor associated macrophages [56]. The present data thus support the notion that NLCs represent tumor associated macrophages (TAM) characterized by an immunosuppressive phenotype. In keeping with this suggestion are also the reports from Filip et al., Maffei et al., and Ysebaert et al. [67,68,69] that proposed that NLCs are TAM characterized by deregulation of genes involved in inflammation. It has been further observed that in the Eµ-TCL1 mouse model, representing a widely accepted in vivo model of CLL, myeloid cells, infiltrating the peritoneal cavity, are skewed toward the M2 phenotype: interestingly, their depletion by liposomal clodronate resulted in a significant control of disease development [70]. Additional confirmations of the critical survival support of CLL cells by TAMs come also from the study of Galletti et al. These authors, by the use of a CLL/xenograft model, have demonstrated that targeting of mono/macrophages by either CSFR1 (colony-stimulating factor-1 receptor) or liposomal clodronate markedly inhibited leukemia development further restoring an anti-leukemic phenotype in accessory cells of the microenvironment [71,72]. In addition, it has been reported that Lyn deficiency in μTCL1 mouse model significantly hinders leukemogenesis in peripheral blood and lymphoid organs: in particular Lyn deficient macrophages were significantly less efficient in supporting CLL cells survival. [73]. 

**Figure 2:**
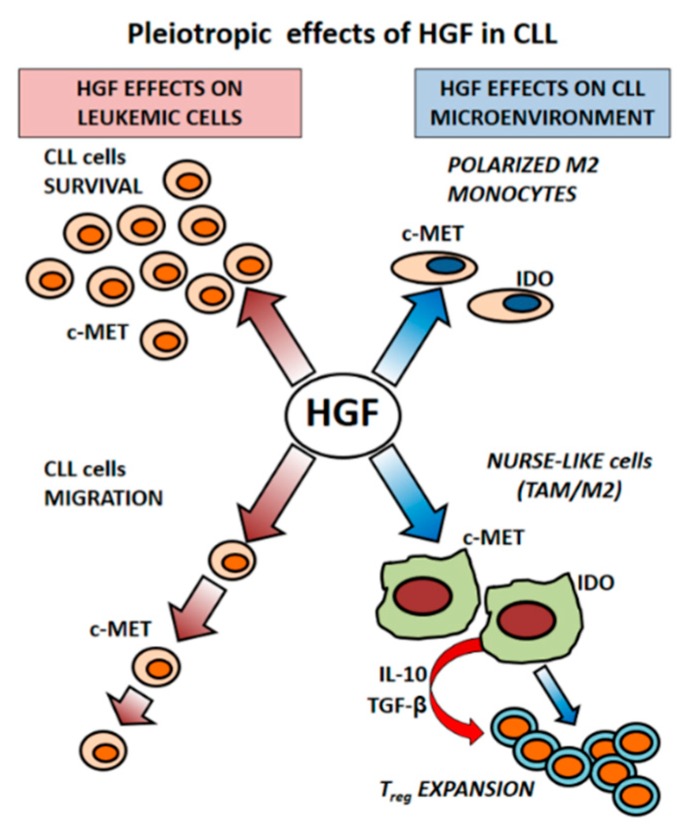
Hypothetical pleiotropic activity of HGF in CLL: HGF enhances viability of CLL cells c-MET+ and stimulates also their migration (personal observation). In addition, HGF affects differentiation of accessory cells of the microenvironment: HGF in fact polarizes monocytes toward an alternative immunosuppressive M2 phenotype by increasing the expression of IDO and c-MET on their membrane. M2 monocytes and nurse-like cells, tumor associated macrophages typically present in CLL, in turn, stimulate expansion of T regulatory cells through the production of IL-10 TGF-beta and IDO expression.

IDO degrades trypthophan into kynurinine which, in turn, inhibits effector T cells and stimulates Treg expansion. It is of interest to note that kynurinine–tryptophan ratios, reflecting increased IDO activity, have been found higher than normal in sera from CLL patients [74]. In addition the presence of CD4+CD25+FOXp3+ Treg cells was also higher in CLL patients than in normal controls [75,76,77,78]. We have demonstrated that the percentage of CD4+CD25+FOXp3+ T lymphocytes was progressively increased during long term co-cultures of enriched CLL-monocytes with autologous peripheral mononuclear cells from CLL patients, peaking at 8–14 days. It was of further interest to observe that, while CLL cells and NLCs do not constitutively produce HGF, they start to release it when they are co-cultured together: a significant amount of HGF (range: 400–600 pg/mL) was in fact detected in media from co-cultures and we confirmed, by cytofluorimetric analysis, the presence of intracytoplasmic HGF in both CLL cells and NLCs [56]. This finding points to a paracrine but also to an autocrine release of HGF in CLL, suggesting a mutual influence of CLL and accessory cells viability through the action of this cytokine (Figure 2). Indeed similar results, showing an increased HGF production, were already reported in MM, relatively to BMSC and malignant plasma cells co-cultured together, both in a cell contact setting or when separated by a permeable membrane [49].

Whether particular soluble factors may be involved in increasing HGF release from leukemic B cells is still unknown. It has been reported that in naïve splenic B cells from mice, HGF, produced after the interaction between CD74 and macrophage migration inhibitory factor (MIF), regulates the survival of these cells [79]. MIF, among others candidate cytokines, could be thus involved in mediating c-MET expression and HGF release by CLL cells or by monocytes. It is of interest to note that high levels of MIF have been detected in sera from CLL patients and that the inhibition of MIF delayed CLL development in the Eµ-TCL1 mouse model crossed with a MIF^−/−^ knockout mouse. Here the authors suggest that MIF sustained the expansion of the CLL clone through interaction with macrophages: the last appeared in fact reduced in the spleen and in BM of the Eµ-TCL1^+/wt^ MIF^−/−^ mice [80].

The presence of elevated MIF levels have been also observed in MM patients and higher concentrations of MIF appear implicated in homing of MM cells to bone marrow and in resistance to chemotherapy [81]. In addition a pro-tumoral role of MIF in MM has been recently suggested. Here MIF, released by malignant plasma cells, stimulates IL-6 and IL-8 production from BMSC only after their co-culture. MIF-induced IL-6 and IL-8 secretion was further inhibited by blocking the MIF receptor CD74 or through the addition of the MIF inhibitor ISO-1 [82]. Results, from our studies in CLL as well as from those reported in MM, highlight how neoplastic B cells are capable of shaping their microenvironment thus finally creating a permissive niche. Many factors could be involved in the complex network of interactions between malignant and stromal cells that could favor this process.

Co-culture systems are in part capable of recapitulating CLL cells/tumor microenvironment interactions. Co-cultures of CLL cells with stromal cells, T cells or transfected-fibroblasts expressing CD40L or CD31 [7,83] have been extensively used to mimic leukemic cells interactions within lymph-nodes, bone marrow, or vascular niches: these systems may allow to discriminate whether different accessory cell types influence survival, proliferation, upregulation of membrane antigens (ZAP-70, CD38, IL-21R, IL-23R) on leukemic cells or release of particular cytokines. However, these conventional two-dimensional cultures present limitations with respect to cell density, availability of contact surface and anchorage among cells, and are often devoid of proper biomechanical stimuli [84] and of relevant structured ECM [85]. Indeed, in this light, the role for ECM as a reservoir of bioactive molecules, including HGF and some of its activating enzymes, has been recently described. Moreover ECM-resident molecules, such as CD44, can interfere with c-MET and participate to overall activation of the HGF/c-MET signaling pathways [86]. Therefore, the implementation of 3D culture systems should be envisaged to better mimic all the cell-to-cell/cell-to-ECM relationships that take place in native tissues. Mimicking of a 3D cell niche has been revealed also useful to maintain stem properties of pluripotent stem cells [87]: interestingly c-MET has been indicated as a marker of normal (liver, mammary gland, nervous tissue, gut epithelium) and cancer (breast, brain, colon, pancreas, AML) stem cells [88]. Proper 3D modeling should thus be taken into account trying to identify leukemic cells progenitors in hematological diseases such as MM and CLL. In addition, 3D culture systems should represent a parallel approach to the genetically modified animal models. As a matter of fact, targeted disruption of HGF or c-MET genes have provided relevant insights on their indispensable role in mammalian development; nonetheless KO mice are embryonically lethal. Therefore HGF^−/−^ and c-MET^−/−^ exploitation in a whole living organism is limited unless targeting c-MET deletion in specific tissues through conditional KO mice [89].

Finally, a better comprehension of the complex cross-talk among leukemic cells, stromal cells, and immune cells within a structured model of culture should further clarify the potential role of HGF/c-MET axis in pathogenesis and progression of hematological disease.

## 4. Therapeutic Options to Counteract HGF- and c-MET-Related Cancer Effects

**Figure 3:**
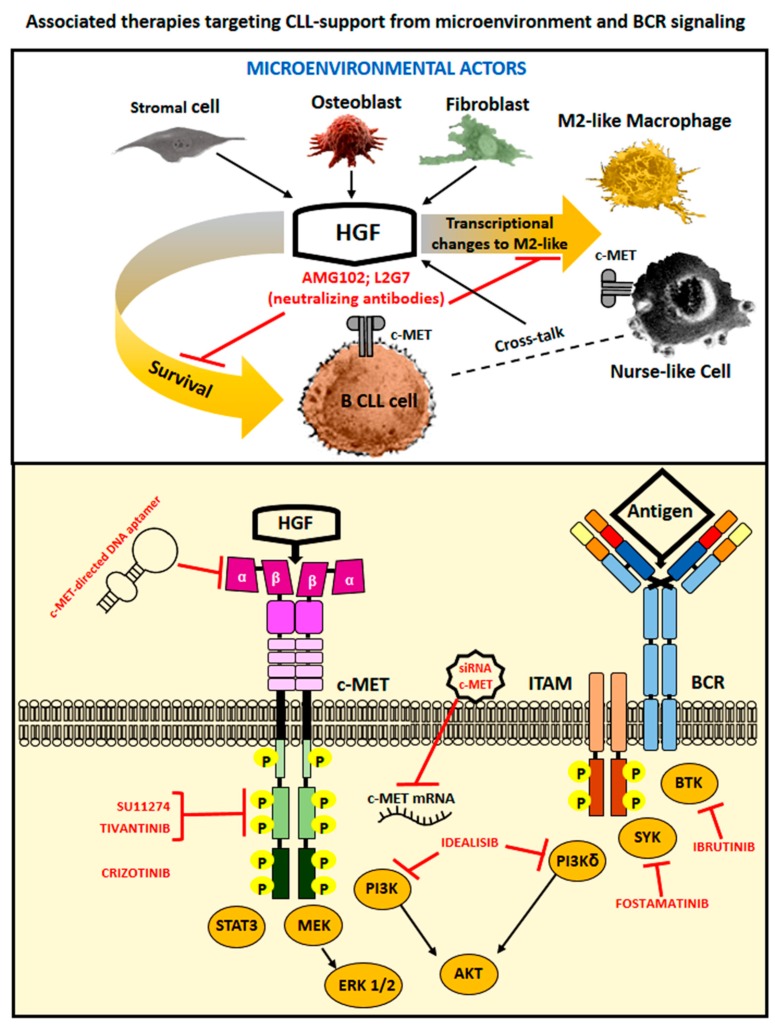
Hypothetical model of associated therapies targeting CLL-support from microenvironment and BCR signaling: HGF released in paracrine and autocrine manner within the CLL microenvironment maintains viability of accessory cells (stromal, osteoblasts cancer associated fibroblasts, and nurse-like cells) which in turn support viability of leukemic cells. The usage of anti-HGF moAb (i.e., L2G7, AMG102), should block CLL support by cells of the microenvironment. On the other side inhibitors of the tyrosine kinase c-MET (i.e., SU11274, Crizotinib, Tivantinib), c-MET siRNA transfected exosomes or a c-MET directed aptamer, in association with inhibitors of tyrosine kinases triggered by BCR (PI3Kd, SYK, BTK), may directly interfere with survival and expansion of the leukemic B cell clone promoted by the inner cell signaling pathways.

The tumor microenvironment has been recognized as a key contributor in progression of neoplastic diseases. Inhibition of c-MET signaling may impair cancer cell growth and at the same time stimulate immune responses via relieving HGF/c-MET induced immunosuppressive effects on mono-macrophages and on T cells. We demonstrated that the c-MET tyrosine kinase inhibitor SU11274 or the neutralizing anti-HGF moAb L2G7 (a kind gift from Galaxy Biotech, Sunnyvale, CA 94089, USA) counteracted increased CLL cells viability induced by HGF treatment or after co-culturing leukemic cells with specific stromal cells (BMSC, trabecular bone cells, the osteoblasto-like cell line MG63 [21] and Figure 1). Our experimental results may thus suggest that c-MET tyrosine kinase inhibitors could be successfully utilized to induce CLL cells death as well as to block CLL/accessory cells interactions that support survival of the leukemic B cell clone (Figure 3). Indeed, tumor microenvironment participates in resistance to molecular-targeted drugs as well as in neoplastic cells spreading: the role of HGF/c-MET axis activation in these processes has been often demonstrated and therapeutically tackled in solid tumors [11], while only a limited number of studies has instead exploited this possibility in hematological neoplasms. In addition most of the studies are limited to in vitro assays with cell lines. The requirement for HGF/c-MET signaling for autonomous cell growth in vitro has been shown for tree AML cell lines (HEL, SKNO, KG1): treatment with the tyrosine kinase inhibitor SU11274 (1 µM) in fact induced cell death. Moreover the treatment with Crizotinib (PF2341066: c-MET/ALK inhibitor; 0.1 µM) led to decreased colony formation of HGF-expressing primary AML samples [25]. Also the growth of Primary Effusion Lymphoma (PEL) was suppressed by the use of Crizotinib in vitro (concentration range 0.05 to 1.6 µM) as well as in vivo, in a NOD/SCID mice pre-clinical model [31]. A phase II study with the c-MET inhibitor Tivantinib has been recently reported in patients with relapsed/refractory multiple myeloma [90]. Among 11 patients treated with Tivantinib, only 4 (36%) showed stable disease as the best response, while the remainder showed disease progression. Although this study may not seem too much promising to cure MM by the use of Tivantinib as a single agent, the authors here suggest that it could represent an attractive option if combined with other anti-myeloma drugs such as proteasome inhibitors, since c-MET activation is often associated with clinical drug resistance in MM [91]. Recently it has been described that the DNA single stranded aptamer SL1 shows a high affinity and specificity for c-MET. SL1 (1–4 µM) was capable of selectively bind to c-MET+ MM cells but not to normal B cells thus suppressing growth, adhesion, migration, and downstream c-MET signaling (pERK, pAKT) of MM cells in a co-culture model with the HS5 cell line. SL1 further appeared anti-proliferative in primary CD138+ MM cells and capable of acting in synergy with Bortezomib (5–7.5 µM) [92]. A recent study has further reported that the histone deacetylase 6 (HDAC6) plays an oncogenic role in DLBCL via indirect activation of c-MET signaling: the efficacy of a combined therapy with Ricolinostat (HDAC6 inhibitor: 1–2.5 µM) and Crizotinib (13–75 µM) was here demonstrated. This combinatorial therapy could block the growth of DLBCL cells in vitro as well as in a xenograft model developed in mice [93]. 

The utilization of mesenchymal-derived exosomes further represents today a novel and emerging alternative chance of intervention to contrast cancer-supportive microenvironments [94]. Exosomes are 30–120 nm membrane bound vesicles secreted naturally by almost all cells, both in physiological and in pathological conditions. Exosomes, recently discovered and described as an inter-cells-communication way, contain proteins, lipids, DNA, mRNA, miRNAs and long noncoding RNAs which can be transferred from producer to recipient cells. Exosomes released by cancer cells are capable of subverting the physiological functional program of microenvironmental cells [95]. On the other hand exosomes derived from normal mesenchymal cells have been demonstrated useful in several experimental assets: to repair damaged tissues, to modulate differentiation and function of lymphocytes/monocytes, to inhibit cancer proliferation and dissemination [96,97,98,99,100]. Interestingly, the activation of the HGF/c-MET axis may be involved in pro-tumoral microenvironment changes caused by cancer cells-released exosomes. For example, exosomes from melanoma could induce an increase of c-MET activation in cells derived from BM, thus reprogramming them toward a pro-angiogenic phenotype. It is further worth noting that the administration of exosomes with high c-MET levels facilitated metastasis of melanoma cells exhibiting lower metastatic ability [11,101,102]. Moreover, a recent paper has described the possibility of utilizing exosomes derived from BMSC of healthy controls to suppress myeloma-related angiogenesis [103]. Here, Tomoiro et al. have first observed that anti-angiogenic effects induced by exosomes-derived BMSC appeared age-related. Exosomes from young donors were in fact more efficient than those from older individuals due to higher levels of expression of miRNA340 which acted as the mediator of the observed anti-angiogenic effect. Through the subsequent transfection of miRNA340 in older BMSC exosomes, the authors could then demonstrate a significant inhibition of the growth of MM cell lines or of CD138+ primary MM cells in an in vivo BALB/c nude mice model. Interestingly anti-angiogenic effects mediated by exosomes carrying miRNA340 have been related to inhibition of the HGF/c-MET signaling pathways: HUVEC treated with miRNA340 exosomes showed a 60% reduction of c-MET expression and the endothelial tube formation detected in an in vitro assay in matrigel appeared also consistently suppressed [103]. Finally, we would like to suggest that, based on results from our studies and from other authors’, therapies targeting the HGF/c-MET interaction could be of particular interest in counteracting microenvironment–driven support to leukemic cells: combinatorial therapies utilizing conventional drugs in association with c-MET kinase inhibitors or exosomes-delivered effector molecules could thus effectively implement therapeutic approaches actually in use (Figure 3).

## 5. Concluding Remarks

The tumor micro-environment has a pivotal role in promoting tumor development. Stromal cells, integral components of the tumor microenvironment, activate c-MET through HGF release. Therefore, high c-MET expression and involvement of HGF/c-MET pathway has been observed in a large part of cancer and has been correlated with poor patient survival. Abnormal activation of c-MET tyrosine kinase is further critical for resistance to targeted therapies. Although studies referring to activation of the HGF/c-MET axis in hematological diseases are relatively a few, it appears that this interaction may, directly or indirectly, favor the expansion of the leukemic clone. We in particular described a double effect of HGF leading to enhanced survival of CLL cells but also driving monocytes/macrophages toward an alternative M2 suppressor phenotype, thus facilitating tumor evasion from immune control. Our findings may thus indicate that targeting of HGF, a novel microenvironmental factor involved in CLL pathogenesis, could improve conventional therapies to cure this disease by inhibiting leukemic cell growth and by restoring specific immune responses against the tumor.

## Figures and Tables

**Table 1:** c-MET/HGF expression on tumor cells and auto/paracrine activation of this pathway.

Neoplasia of Mature B Cells	Neoplastic Cells			c-MET Activation		Refs
	c-MET	HGF	HGFA	Autocrine	Paracrine	
Mantle Cell Lymphoma	−	−	n.d.	−	−	[34]
Marginal Zone Lymphoma	−	−	n.d.	−	−	[34]
Follicular Lymphoma	+/−	−	n.d.	−	+	[34]
Diffuse Large B Cell Lymphoma	+	−	+	−	+	[34,35]
Chronic Lymphocytic Leukemia	+/−	+/−	+/−	+	+	[21,34,56]
Burkitt Lymphoma	+/−	−	n.d.	−	+	[30,33,34]
Primary Effusion Lymphoma	+	+	+	+	+	[30]
Multiple Myeloma	+	+	+	+	+	[47,48,50]
Hodgkin Lymphoma	+	−	n.d.	−	+	[51,52,53]

For c-MET, HGF, HGFA: +, − or +/−: high, negative or intermediate expression levels; for c-MET activation: + or − indicate an ascertained modality of receptor activation; n.d.: not determined.

## References

[1] Chiorazzi N., Rai K.R., Ferrarini M. (2005). Chronic lymphocytic leukemia. N. Engl. J. Med..

[2] Burger J.A., O’Brien S. (2018). Evolution of CLL treatment—From chemoimmunotherapy to targeted and individualized therapy. Nat. Rev. Clin. Oncol..

[3] Vitale C., Burger J.A. (2016). Chronic lymphocytic leukemia therapy: New targeted therapies on the way. Expert Opin. Pharm..

[4] Burger J.A., Ghia P., Rosenwald A., Caligaris-Cappio F. (2009). The microenvironment in mature B-cell malignancies: A target for new treatment strategies. Blood.

[5] Caligaris-Cappio F. (2003). Role of the microenvironment in chronic lymphocytic leukaemia. Br. J. Haematol..

[6] Cutrona G., Tripodo C., Matis S., Recchia A.G., Massucco C., Fabbi M., Colombo M., Emionite L., Sangaletti S., Gulino A. (2018). Microenvironmental regulation of the IL-23R/IL-23 axis overrides chronic lymphocytic leukemia indolence. Sci. Transl. Med..

[7] De Totero D., Meazza R., Capaia M., Fabbi M., Azzarone B., Balleari E., Gobbi M., Cutrona G., Ferrarini M., Ferrini S. (2008). The opposite effects of IL-15 and IL-21 on CLL B cells correlate with differential activation of the JAK/STAT and ERK1/2 pathways. Blood.

[8] Giannoni P., de Totero D., Atta-ur-Rahman (2014). Is a protective microenvironment the Achille’s heel in chronic lymphocytic leukemia?. Frontiers in Clinical Drug Research-Hematology.

[9] Lagneaux L., Delforge A., Bron D., De Bruyn C., Stryckmans P. (1998). Chronic lymphocytic leukemic B cells but not normal B cells are rescued from apoptosis by contact with normal bone marrow stromal cells. Blood.

[10] Birchmeier C., Birchmeier W., Gherardi E., Vande Woude G.F. (2003). Met, metastasis, motility and more. Nat. Rev. Mol. Cell. Biol..

[11] Matsumoto K., Umitsu M., De Silva D.M., Roy A., Bottaro D.P. (2017). Hepatocyte growth factor/MET in cancer progression and biomarker discovery. Cancer Sci..

[12] Nakamura T., Mizuno S. (2010). The discovery of hepatocyte growth factor (HGF) and its significance for cell biology, life sciences and clinical medicine. Proc. Jpn. Acad. Ser. B Phys. Biol. Sci..

[13] Trusolino L., Bertotti A., Comoglio P.M. (2010). MET signalling: Principles and functions in development, organ regeneration and cancer. Nat. Rev. Mol. Cell. Biol..

[14] Lam B.Q., Dai L., Qin Z. (2016). The role of HGF/c-MET signaling pathway in lymphoma. J. Hematol. Oncol..

[15] Mahtouk K., Tjin E.P., Spaargaren M., Pals S.T. (2010). The HGF/MET pathway as target for the treatment of multiple myeloma and B-cell lymphomas. Biochim. Biophys. Acta.

[16] Boissinot M., Vilaine M., Hermouet S. (2014). The Hepatocyte Growth Factor (HGF)/Met Axis: A Neglected Target in the Treatment of Chronic Myeloproliferative Neoplasms?. Cancers.

[17] Ferrucci A., Moschetta M., Frassanito M.A., Berardi S., Catacchio I., Ria R., Racanelli V., Caivano A., Solimando A.G., Vergara D. (2014). A HGF/cMET autocrine loop is operative in multiple myeloma bone marrow endothelial cells and may represent a novel therapeutic target. Clin. Cancer Res..

[18] Gambella M., Palumbo A., Rocci A. (2015). MET/HGF pathway in multiple myeloma: From diagnosis to targeted therapy?. Expert Rev. Mol. Diagnos..

[19] Wader K.F., Fagerli U.M., Holt R.U., Borset M., Sundan A., Waage A. (2011). Soluble c-Met in serum of patients with multiple myeloma: Correlation with clinical parameters. Eur. J. Haematol..

[20] Rocci A., Gambella M., Aschero S., Baldi I., Trusolino L., Cavallo F., Gay F., Larocca A., Magarotto V., Omede P. (2014). MET dysregulation is a hallmark of aggressive disease in multiple myeloma patients. Br. J. Haematol..

[21] Giannoni P., Scaglione S., Quarto R., Narcisi R., Parodi M., Balleari E., Barbieri F., Pattarozzi A., Florio T., Ferrini S. (2011). An interaction between hepatocyte growth factor and its receptor (c-MET) prolongs the survival of chronic lymphocytic leukemic cells through STAT3 phosphorylation: A potential role of mesenchymal cells in the disease. Haematologica.

[22] Gohda E., Tsubouchi H., Nakayama H., Hirono S., Sakiyama O., Takahashi K., Miyazaki H., Hashimoto S., Daikuhara Y. (1988). Purification and partial characterization of hepatocyte growth factor from plasma of a patient with fulminant hepatic failure. J. Clin. Investig..

[23] Jiang W., Hiscox S., Matsumoto K., Nakamura T. (1999). Hepatocyte growth factor/scatter factor, its molecular, cellular and clinical implications in cancer. Crit. Rev. Oncol./Hematol..

[24] Van der Voort R., Taher T.E., Keehnen R.M., Smit L., Groenink M., Pals S.T. (1997). Paracrine regulation of germinal center B cell adhesion through the c-met-hepatocyte growth factor/scatter factor pathway. J. Exp. Med..

[25] Kentsis A., Reed C., Rice K.L., Sanda T., Rodig S.J., Tholouli E., Christie A., Valk P.J., Delwel R., Ngo V. (2012). Autocrine activation of the MET receptor tyrosine kinase in acute myeloid leukemia. Nat. Med..

[26] Majeti R., Becker M.W., Tian Q., Lee T.L., Yan X., Liu R., Chiang J.H., Hood L., Clarke M.F., Weissman I.L. (2009). Dysregulated gene expression networks in human acute myelogenous leukemia stem cells. Proc. Natl. Acad. Sci. USA.

[27] Imaizumi Y., Murota H., Kanda S., Hishikawa Y., Koji T., Taguchi T., Tanaka Y., Yamada Y., Ikeda S., Kohno T. (2003). Expression of the c-Met proto-oncogene and its possible involvement in liver invasion in adult T-cell leukemia. Clin. Cancer Res..

[28] Onimaru Y., Tsukasaki K., Murata K., Imaizumi Y., Choi Y.L., Hasegawa H., Sugahara K., Yamada Y., Hayashi T., Nakashima M. (2008). Autocrine and/or paracrine growth of aggressive ATLL cells caused by HGF and c-Met. Int. J. Oncol..

[29] Kumai T., Matsuda Y., Ohkuri T., Oikawa K., Ishibashi K., Aoki N., Kimura S., Harabuchi Y., Celis E., Kobayashi H. (2015). c-Met is a novel tumor associated antigen for T-cell based immunotherapy against NK/T cell lymphoma. Oncoimmunology.

[30] Capello D., Gaidano G., Gallicchio M., Gloghini A., Medico E., Vivenza D., Buonaiuto D., Fassone L., Avanzi G.C., Saglio G. (2000). The tyrosine kinase receptor met and its ligand HGF are co-expressed and functionally active in HHV-8 positive primary effusion lymphoma. Leukemia.

[31] Dai L., Trillo-Tinoco J., Cao Y., Bonstaff K., Doyle L., Del Valle L., Whitby D., Parsons C., Reiss K., Zabaleta J. (2015). Targeting HGF/c-MET induces cell cycle arrest, DNA damage, and apoptosis for primary effusion lymphoma. Blood.

[32] Lam B.Q., Dai L., Li L., Qiao J., Lin Z., Qin Z. (2017). Molecular mechanisms of activating c-MET in KSHV+ primary effusion lymphoma. Oncotarget.

[33] Jucker M., Gunther A., Gradl G., Fonatsch C., Krueger G., Diehl V., Tesch H. (1994). The Met/hepatocyte growth factor receptor (HGFR) gene is overexpressed in some cases of human leukemia and lymphoma. Leuk. Res..

[34] Tjin E.P., Groen R.W., Vogelzang I., Derksen P.W., Klok M.D., Meijer H.P., van Eeden S., Pals S.T., Spaargaren M. (2006). Functional analysis of HGF/MET signaling and aberrant HGF-activator expression in diffuse large B-cell lymphoma. Blood.

[35] Kawano R., Ohshima K., Karube K., Yamaguchi T., Kohno S., Suzumiya J., Kikuchi M., Tamura K. (2004). Prognostic significance of hepatocyte growth factor and c-MET expression in patients with diffuse large B-cell lymphoma. Br. J. Haematol..

[36] Koh Y.W., Hwang H.S., Jung S.J., Park C., Yoon D.H., Suh C., Huh J. (2013). Receptor tyrosine kinases MET and RON as prognostic factors in diffuse large B-cell lymphoma patients receiving R-CHOP. Cancer Sci..

[37] Uddin S., Hussain A.R., Ahmed M., Al-Dayel F., Bu R., Bavi P., Al-Kuraya K.S. (2010). Inhibition of c-MET is a potential therapeutic strategy for treatment of diffuse large B-cell lymphoma. Lab. Investig..

[38] Bjorck E., Ek S., Landgren O., Jerkeman M., Ehinger M., Bjorkholm M., Borrebaeck C.A., Porwit-MacDonald A., Nordenskjold M. (2005). High expression of cyclin B1 predicts a favorable outcome in patients with follicular lymphoma. Blood.

[39] Elenitoba-Johnson K.S., Jenson S.D., Abbott R.T., Palais R.A., Bohling S.D., Lin Z., Tripp S., Shami P.J., Wang L.Y., Coupland R.W. (2003). Involvement of multiple signaling pathways in follicular lymphoma transformation: p38-mitogen-activated protein kinase as a target for therapy. Proc. Natl. Acad. Sci. USA.

[40] Giles F.J., Vose J.M., Do K.A., Johnson M.M., Manshouri T., Bociek G., Bierman P.J., O’Brien S.M., Kantarjian H.M., Armitage J.O. (2004). Clinical relevance of circulating angiogenic factors in patients with non-Hodgkin’s lymphoma or Hodgkin’s lymphoma. Leuk. Res..

[41] Hsiao L.T., Lin J.T., Yu I.T., Chiou T.J., Liu J.H., Yen C.C., Wang W.S., Chen P.M. (2003). High serum hepatocyte growth factor level in patients with non-Hodgkin’s lymphoma. Eur. J. Haematol..

[42] Seidel C., Borset M., Turesson I., Abildgaard N., Sundan A., Waage A. (1998). Elevated serum concentrations of hepatocyte growth factor in patients with multiple myeloma. The Nordic Myeloma Study Group. Blood.

[43] Wader K.F., Fagerli U.M., Holt R.U., Stordal B., Borset M., Sundan A., Waage A. (2008). Elevated serum concentrations of activated hepatocyte growth factor activator in patients with multiple myeloma. Eur. J. Haematol..

[44] Pour L., Svachova H., Adam Z., Almasi M., Buresova L., Buchler T., Kovarova L., Nemec P., Penka M., Vorlicek J. (2010). Levels of angiogenic factors in patients with multiple myeloma correlate with treatment response. Ann. Hematol..

[45] Kristensen I.B., Christensen J.H., Lyng M.B., Moller M.B., Pedersen L., Rasmussen L.M., Ditzel H.J., Abildgaard N. (2013). Hepatocyte growth factor pathway upregulation in the bone marrow microenvironment in multiple myeloma is associated with lytic bone disease. Br. J. Haematol..

[46] Standal T., Abildgaard N., Fagerli U.M., Stordal B., Hjertner O., Borset M., Sundan A. (2007). HGF inhibits BMP-induced osteoblastogenesis: Possible implications for the bone disease of multiple myeloma. Blood.

[47] Borset M., Hjorth-Hansen H., Seidel C., Sundan A., Waage A. (1996). Hepatocyte growth factor and its receptor c-met in multiple myeloma. Blood.

[48] Borset M., Lien E., Espevik T., Helseth E., Waage A., Sundan A. (1996). Concomitant expression of hepatocyte growth factor/scatter factor and the receptor c-MET in human myeloma cell lines. J. Biol. Chem..

[49] Rampa C., Tian E., Vatsveen T.K., Buene G., Slordahl T.S., Borset M., Waage A., Sundan A. (2014). Identification of the source of elevated hepatocyte growth factor levels in multiple myeloma patients. Biomark. Res..

[50] Wader K.F., Fagerli U.M., Borset M., Lydersen S., Hov H., Sundan A., Bofin A., Waage A. (2012). Immunohistochemical analysis of hepatocyte growth factor and c-Met in plasma cell disease. Histopathology.

[51] Pons E., Uphoff C.C., Drexler H.G. (1998). Expression of hepatocyte growth factor and its receptor c-met in human leukemia-lymphoma cell lines. Leuk. Res..

[52] Teofili L., Di Febo A.L., Pierconti F., Maggiano N., Bendandi M., Rutella S., Cingolani A., Di Renzo N., Musto P., Pileri S. (2001). Expression of the c-met proto-oncogene and its ligand, hepatocyte growth factor, in Hodgkin disease. Blood.

[53] Weimar I.S., de Jong D., Muller E.J., Nakamura T., van Gorp J.M., de Gast G.C., Gerritsen W.R. (1997). Hepatocyte growth factor/scatter factor promotes adhesion of lymphoma cells to extracellular matrix molecules via alpha 4 beta 1 and alpha 5 beta 1 integrins. Blood.

[54] Bedewy M., El-Maghraby S., Bedewy A. (2013). CD163 and c-Met expression in the lymph node and the correlations between elevated levels of serum free light chain and the different clinicopathological parameters of advanced classical Hodgkin’s lymphoma. Blood Res..

[55] Xu C., Plattel W., van den Berg A., Ruther N., Huang X., Wang M., de Jong D., Vos H., van Imhoff G., Viardot A. (2012). Expression of the c-Met oncogene by tumor cells predicts a favorable outcome in classical Hodgkin’s lymphoma. Haematologica.

[56] Giannoni P., Pietra G., Travaini G., Quarto R., Shyti G., Benelli R., Ottaggio L., Mingari M.C., Zupo S., Cutrona G. (2014). Chronic Lymphocytic Leukemia Nurse-like cells express the hepatocyte growth factor receptor (c-MET) and indoleamine 2,3-dioxygenase and display features of immunosuppressive type 2 skewed macrophages. Haematologica.

[57] Messmer B.T., Messmer D., Allen S.L., Kolitz J.E., Kudalkar P., Cesar D., Murphy E.J., Koduru P., Ferrarini M., Zupo S. (2005). In vivo measurements document the dynamic cellular kinetics of chronic lymphocytic leukemia B cells. J. Clin. Investig..

[58] Herishanu Y., Perez-Galan P., Liu D., Biancotto A., Pittaluga S., Vire B., Gibellini F., Njuguna N., Lee E., Stennett L. (2011). The lymph node microenvironment promotes B-cell receptor signaling, NF-kappaB activation, and tumor proliferation in chronic lymphocytic leukemia. Blood.

[59] Herndon T.M., Chen S.S., Saba N.S., Valdez J., Emson C., Gatmaitan M., Tian X., Hughes T.E., Sun C., Arthur D.C. (2017). Direct in vivo evidence for increased proliferation of CLL cells in lymph nodes compared to bone marrow and peripheral blood. Leukemia.

[60] Eksioglu-Demiralp E., Akdeniz T., Bayik M. (2010). Aberrant expression of c-met and HGF/c-met pathway provides survival advantage in B-chronic lymphocytic leukemia. Cytom. Part B Clin. Cytom..

[61] Aguayo A., Kantarjian H., Manshouri T., Gidel C., Estey E., Thomas D., Koller C., Estrov Z., O’Brien S., Keating M. (2000). Angiogenesis in acute and chronic leukemias and myelodysplastic syndromes. Blood.

[62] Chen P.M., Liu K.J., Hsu P.J., Wei C.F., Bai C.H., Ho L.J., Sytwu H.K., Yen B.L. (2014). Induction of immunomodulatory monocytes by human mesenchymal stem cell-derived hepatocyte growth factor through ERK1/2. J. Leukoc Biol..

[63] Rutella S., Bonanno G., Procoli A., Mariotti A., de Ritis D.G., Curti A., Danese S., Pessina G., Pandolfi S., Natoni F. (2006). Hepatocyte growth factor favors monocyte differentiation into regulatory interleukin (IL)-10++IL-12low/neg accessory cells with dendritic-cell features. Blood.

[64] Burger J.A., Tsukada N., Burger M., Zvaifler N.J., Dell’Aquila M., Kipps T.J. (2000). Blood-derived nurse-like cells protect chronic lymphocytic leukemia B cells from spontaneous apoptosis through stromal cell-derived factor-1. Blood.

[65] Tsukada N., Burger J.A., Zvaifler N.J., Kipps T.J. (2002). Distinctive features of “nurselike” cells that differentiate in the context of chronic lymphocytic leukemia. Blood.

[66] Bhattacharya N., Diener S., Idler I.S., Rauen J., Habe S., Busch H., Habermann A., Zenz T., Dohner H., Stilgenbauer S. (2011). Nurse-like cells show deregulated expression of genes involved in immunocompetence. Br. J. Haematol..

[67] Filip A.A., Cisel B., Koczkodaj D., Wasik-Szczepanek E., Piersiak T., Dmoszynska A. (2013). Circulating microenvironment of CLL: Are nurse-like cells related to tumor-associated macrophages?. Blood Cells Mol. Dis..

[68] Maffei R., Bulgarelli J., Fiorcari S., Bertoncelli L., Martinelli S., Guarnotta C., Castelli I., Deaglio S., Debbia G., De Biasi S. (2013). The monocytic population in chronic lymphocytic leukemia shows altered composition and deregulation of genes involved in phagocytosis and inflammation. Haematologica.

[69] Ysebaert L., Fournie J.J. (2011). Genomic and phenotypic characterization of nurse-like cells that promote drug resistance in chronic lymphocytic leukemia. Leuk. Lymphoma.

[70] Hanna B.S., McClanahan F., Yazdanparast H., Zaborsky N., Kalter V., Rossner P.M., Benner A., Durr C., Egle A., Gribben J.G. (2016). Depletion of CLL-associated patrolling monocytes and macrophages controls disease development and repairs immune dysfunction in vivo. Leukemia.

[71] Galletti G., Caligaris-Cappio F., Bertilaccio M.T. (2016). B cells and macrophages pursue a common path toward the development and progression of chronic lymphocytic leukemia. Leukemia.

[72] Galletti G., Scielzo C., Barbaglio F., Rodriguez T.V., Riba M., Lazarevic D., Cittaro D., Simonetti G., Ranghetti P., Scarfo L. (2016). Targeting Macrophages Sensitizes Chronic Lymphocytic Leukemia to Apoptosis and Inhibits Disease Progression. Cell Rep..

[73] Nguyen P.H., Fedorchenko O., Rosen N., Koch M., Barthel R., Winarski T., Florin A., Wunderlich F.T., Reinart N., Hallek M. (2016). LYN Kinase in the Tumor Microenvironment Is Essential for the Progression of Chronic Lymphocytic Leukemia. Cancer Cell.

[74] Lindstrom V., Aittoniemi J., Jylhava J., Eklund C., Hurme M., Paavonen T., Oja S.S., Itala-Remes M., Sinisalo M. (2012). Indoleamine 2,3-dioxygenase activity and expression in patients with chronic lymphocytic leukemia. Clin. Lymphoma Myeloma Leuk..

[75] D'Arena G., Laurenti L., Minervini M.M., Deaglio S., Bonello L., De Martino L., De Padua L., Savino L., Tarnani M., De Feo V. (2011). Regulatory T-cell number is increased in chronic lymphocytic leukemia patients and correlates with progressive disease. Leuk. Res..

[76] Giannopoulos K., Schmitt M., Kowal M., Wlasiuk P., Bojarska-Junak A., Chen J., Rolinski J., Dmoszynska A. (2008). Characterization of regulatory T cells in patients with B-cell chronic lymphocytic leukemia. Oncol. Rep..

[77] Jadidi-Niaragh F., Yousefi M., Memarian A., Hojjat-Farsangi M., Khoshnoodi J., Razavi S.M., Jeddi-Tehrani M., Shokri F. (2013). Increased frequency of CD8+ and CD4+ regulatory T cells in chronic lymphocytic leukemia: Association with disease progression. Cancer Investig..

[78] Jak M., Mous R., Remmerswaal E.B., Spijker R., Jaspers A., Yague A., Eldering E., Van Lier R.A., Van Oers M.H. (2009). Enhanced formation and survival of CD4+ CD25hi Foxp3+ T-cells in chronic lymphocytic leukemia. Leuk. Lymphoma.

[79] Gordin M., Tesio M., Cohen S., Gore Y., Lantner F., Leng L., Bucala R., Shachar I. (2010). c-Met and its ligand hepatocyte growth factor/scatter factor regulate mature B cell survival in a pathway induced by CD74. J. Immunol..

[80] Reinart N., Nguyen P.H., Boucas J., Rosen N., Kvasnicka H.M., Heukamp L., Rudolph C., Ristovska V., Velmans T., Mueller C. (2013). Delayed development of chronic lymphocytic leukemia in the absence of macrophage migration inhibitory factor. Blood.

[81] Zheng Y., Wang Q., Li T., Qian J., Lu Y., Li Y., Bi E., Reu F., Qin Y., Drazba J. (2016). Role of Myeloma-Derived MIF in Myeloma Cell Adhesion to Bone Marrow and Chemotherapy Response. J. Natl. Cancer Inst..

[82] Piddock R.E., Marlein C.R., Abdul-Aziz A., Shafat M.S., Auger M.J., Bowles K.M., Rushworth S.A. (2018). Myeloma-derived macrophage inhibitory factor regulates bone marrow stromal cell-derived IL-6 via c-MYC. J. Hematol. Oncol..

[83] Hamilton E., Pearce L., Morgan L., Robinson S., Ware V., Brennan P., Thomas N.S., Yallop D., Devereux S., Fegan C. (2012). Mimicking the tumour microenvironment: Three different co-culture systems induce a similar phenotype but distinct proliferative signals in primary chronic lymphocytic leukaemia cells. Br. J. Haematol..

[84] Marrella A., Giannoni P., Pulsoni I., Quarto R., Raiteri R., Scaglione S. (2018). Topographical Features of Graphene-Oxide-Functionalized Substrates Modulate Cancer and Healthy Cell Adhesion Based on the Cell Tissue of Origin. ACS Appl. Mater. Interfaces.

[85] Sangaletti S., Chiodoni C., Tripodo C., Colombo M.P. (2017). The good and bad of targeting cancer-associated extracellular matrix. Curr. Opin. Pharmacol..

[86] Noriega-Guerra H., Freitas V.M. (2018). Extracellular Matrix Influencing HGF/c-MET Signaling Pathway: Impact on Cancer Progression. Int. J. Mol. Sci..

[87] Lou Y.R., Kanninen L., Kaehr B., Townson J.L., Niklander J., Harjumaki R., Jeffrey Brinker C., Yliperttula M. (2015). Silica bioreplication preserves three-dimensional spheroid structures of human pluripotent stem cells and HepG2 cells. Sci. Rep..

[88] Comoglio P.M., Trusolino L., Boccaccio C. (2018). Known and novel roles of the MET oncogene in cancer: A coherent approach to targeted therapy. Nat. Rev. Cancer.

[89] Kato T. (2017). Biological roles of hepatocyte growth factor-Met signaling from genetically modified animals. Biomed. Rep..

[90] Baljevic M., Zaman S., Baladandayuthapani V., Lin Y.H., de Partovi C.M., Berkova Z., Amini B., Thomas S.K., Shah J.J., Weber D.M. (2017). Phase II study of the c-MET inhibitor tivantinib (ARQ 197) in patients with relapsed or relapsed/refractory multiple myeloma. Ann. Hematol..

[91] Moschetta M., Basile A., Ferrucci A., Frassanito M.A., Rao L., Ria R., Solimando A.G., Giuliani N., Boccarelli A., Fumarola F. (2013). Novel targeting of phospho-cMET overcomes drug resistance and induces antitumor activity in multiple myeloma. Clin. Cancer Res..

[92] Zhang Y., Gao H., Zhou W., Sun S., Zeng Y., Zhang H., Liang L., Xiao X., Song J., Ye M. (2018). Targeting c-met receptor tyrosine kinase by the DNA aptamer SL1 as a potential novel therapeutic option for myeloma. J. Cell. Mol. Med..

[93] Liu Z., Cai Y., Yang Y., Li A., Bi R., Wang L., Shen X., Wang W., Jia Y., Yu B. (2018). Activation of MET signaling by HDAC6 offers a rationale for a novel ricolinostat and crizotinib combinatorial therapeutic strategy in diffuse large B-cell lymphoma. J. Pathol..

[94] Wu K., Xing F., Wu S.Y., Watabe K. (2017). Extracellular vesicles as emerging targets in cancer: Recent development from bench to bedside. Biochim. Biophys. Acta Rev. Cancer.

[95] Yu S., Cao H., Shen B., Feng J. (2015). Tumor-derived exosomes in cancer progression and treatment failure. Oncotarget.

[96] Lai R.C., Chen T.S., Lim S.K. (2011). Mesenchymal stem cell exosome: A novel stem cell-based therapy for cardiovascular disease. Regener. Med..

[97] Sdrimas K., Kourembanas S. (2014). MSC microvesicles for the treatment of lung disease: A new paradigm for cell-free therapy. Antioxid. Redox signal..

[98] Zhu Y.G., Feng X.M., Abbott J., Fang X.H., Hao Q., Monsel A., Qu J.M., Matthay M.A., Lee J.W. (2014). Human mesenchymal stem cell microvesicles for treatment of Escherichia coli endotoxin-induced acute lung injury in mice. Stem Cells.

[99] Riazifar M., Pone E.J., Lotvall J., Zhao W. (2017). Stem Cell Extracellular Vesicles: Extended Messages of Regeneration. Annu. Rev. Pharmacol. Toxicol..

[100] Zhou J., Tan X., Tan Y., Li Q., Ma J., Wang G. (2018). Mesenchymal Stem Cell Derived Exosomes in Cancer Progression, Metastasis and Drug Delivery: A Comprehensive Review. J. Cancer.

[101] Adachi E., Sakai K., Nishiuchi T., Imamura R., Sato H., Matsumoto K. (2016). Different growth and metastatic phenotypes associated with a cell-intrinsic change of Met in metastatic melanoma. Oncotarget.

[102] Peinado H., Aleckovic M., Lavotshkin S., Matei I., Costa-Silva B., Moreno-Bueno G., Hergueta-Redondo M., Williams C., Garcia-Santos G., Ghajar C. (2012). Melanoma exosomes educate bone marrow progenitor cells toward a pro-metastatic phenotype through MET. Nat. Med..

[103] Umezu T., Imanishi S., Azuma K., Kobayashi C., Yoshizawa S., Ohyashiki K., Ohyashiki J.H. (2017). Replenishing exosomes from older bone marrow stromal cells with miR-340 inhibits myeloma-related angiogenesis. Blood Adv..

